# The Neuropathology of Autism

**DOI:** 10.6064/2012/703675

**Published:** 2012-12-19

**Authors:** Gene J. Blatt

**Affiliations:** Department of Anatomy & Neurobiology, School of Medicine, Boston University, 72 East Concord Street L 1004, Boston, MA 02118, USA

## Abstract

Autism is a behaviorally defined neurodevelopmental disorder that affects over 1% of new births in the United States and about 2% of boys. The etiologies are unknown and they are genetically complex. There may be epigenetic effects, environmental influences, and other factors that contribute to the mechanisms and affected neural pathway(s). The underlying neuropathology of the disorder has been evolving in the literature to include specific brain areas in the cerebellum, limbic system, and cortex. Part(s) of structures appear to be affected most rather than the entire structure, for example, select nuclei of the amygdala, the fusiform face area, and so forth. Altered cortical organization characterized by more frequent and narrower minicolumns and early overgrowth of the frontal portion of the brain, affects connectivity. Abnormalities include cytoarchitectonic laminar differences, excess white matter neurons, decreased numbers of GABAergic cerebellar Purkinje cells, and other events that can be traced developmentally and cause anomalies in circuitry. Problems with neurotransmission are evident by recent receptor and binding site studies especially in the inhibitory GABA system likely contributing to an imbalance of excitatory/inhibitory transmission. As postmortem findings are related to core behavior symptoms, and technology improves, researchers are gaining a much better perspective of contributing factors to the disorder.

## 1. Introduction

Autism is a behaviorally defined life-long developmental syndrome [1] with a neurobiological basis that includes cellular and structural abnormalities in a variety of brain regions [2–6]. There are deficits in at least 3 domains including sociability, language and range of interests and activities [7–9]. Up until 2012, the diagnostic criteria for autism have relied on the Diagnostic and Statistical Manual of Mental Disorders (DSM-IV-TR), Fourth Edition, Text Revision [10] that placed Autism Spectrum Disorders (ASD) in the category of pervasive developmental disorders (PDD) and also includes two non-ASD PDDs, Rett's syndrome and childhood disintegrative disorders. DSM-IV-TR includes twelve possible symptoms of ASD within the three domains that lead to three possible diagnoses including classical infantile autism (Autistic disorder), higher functioning Asperger's disorder, and Pervasive Developmental Disorder Not Otherwise Specified (PDD-NOS). Autistic disorder requires at least 2 characteristics for the social interaction domain, and one each from communication and repetitive interests and behaviors (RRBs) category present by age 3 years; Asperger's syndrome diagnoses are made if Autistic disorder is ruled out and the patient has at least 2 social deficits and at least one RRB; if both are ruled out then the lesser PDD-NOS diagnosis can be made based on severe social deficits accompanied by impairments in communication or RRBs. The incidence of ASD has been exponentially increasing over the last decade and a recent assessment has reported from 8-year-old children in 2008 that the overall prevalence was 11.3 per 1000, or 1 in 88, and 1 in 54 boys and 1 in 252 girls estimated from 14 Autism and Developmental Disabilities Monitoring (ADDM) Network sites conducted by the Center for Disease Control (CDC) [11]. This increased prevalence rate does not appear to be solely due to better recognition by experts and parents or by wider diagnostic criteria and clearly elevates the status of the developmental disorder from a somewhat rare occurrence as viewed decades ago to a justified urgent public health problem [12]. In 2012, the diagnostic criteria for autism have been updated in a recently proposed DSM-5 that groups affected individuals into a single category, ASD [13]. Recent articles cite variability in how clinical centers will carry out assessments and there is a proposed change to group social interaction with communication domains into a single domain thus reducing the number of criteria [14]. One recent study reported that DSM-5 resulted in 47.79% fewer toddlers being diagnosed with ASD compared to those that were diagnosed with DSM-IV in their study [15]. There is much discussion amongst clinicians regarding the proposed DSM-5, but clearly if it takes effect, has the possibility of reducing the ASD rate due to more stringent criteria in the evaluation requiring five of seven symptoms to be present. The revised criteria could improve the specificity of evaluation but excluding a substantial number of cognitively able individuals especially in the categories other than autistic disorder [16]. 

### 1.1. Neuropathology of Autism Spectrum Disorders (ASD): Affected Brain Areas

An outstanding review article appeared relatively recently by Amaral et al. [17] describing and summarizing brain areas that are affected in autism categorizing them by their functions in autism-related behaviors. To describe in detail every structure that might be impacted in autism is beyond the scope of this paper. However, this paper will focus on the neuropathology of some of the major brain regions that are clearly involved in the disorder and their functional role as they relate to core behavioral domains. It is not meant to be a review of the structural and functional imaging literature which could each be separate treatises. The following sections will discuss brain areas from the hindbrain (cerebellum and inferior olivary complex) and the forebrain (both limbic-hippocampus, entorhinal cortex, amygdala, anterior and posterior cingulate cortex, and neocortical-structural differences including cortical columns and overgrowth, as well as the face processing area in the fusiform gyrus of the temporal lobe). In this way, it will provide an overview of what many in the field consider some of the most pronounced neuropathological and neurotransmitter receptor abnormalities in the ASD brain. 

## 2. Olivocerebellar Abnormalities in ASD 

### 2.1. The Cerebellum in ASD and Reduced Numbers of Purkinje Cells

The cerebellum has long been thought of as an “error correction syste” for fine motor control, balance, and coordination of the body including the spatiotemporal positions and movements of the head and neck. It receives an abundant amount of sensory information including proprioceptive information from muscles and joints, visual, auditory, and somatosensory inputs and sends its outflow via the red nucleus and thalamus (i.e., ventral lateral nucleus and ventral anterior nucleus) to motor cortex. But there has been building evidence over the last two decades that demonstrates that the cerebellum also plays a role in cognition [18–21]. Cerebellar lesions, injuries, and strokes have been shown to produce deficits in verbal abilities and communication, high-order executive functions, and other cognitively related tasks. In fact, cerebellar lesions in 156 patients have been noted to cause altered cognitive patterns including the ability to sequence information, language processing, visuospatial abilities, executive functions, visuospatial memory, and/or attentional problems [22]. Evidence has emerged that there are avenues through which cerebellar outflow can reach other cerebral cortical areas via thalamic nuclei thus enabling the Frontal/Parietal-Cortico-Ponto-Cerebellar-Thalamic-Frontal/Parietal-Cortical connectivity to be complete [23, 24]. One of the most attractive regions of the cerebellum to study is the posterolateral hemispheric region including Crus I and Crus II areas that receive abundant frontopontine projections [25] and also have been shown to have the most numerous Purkinje cell (PC) deficits in the autism brain [3, 26].

There is widespread evidence that the number of cerebellar Purkinje cells is reduced in postmortem brains compared to typically developing controls. An early study reported in four individuals, from 3 to 33 years of age, that 3 had idiopathic autism, two of these with seizure history, and one had autistic-related behaviors showing a generalized reduction of PCs [27]. Another study involved 4 males with no seizure history aged 10–22 years (controls were of age 3–13 years) and found about a 25% decrease in PC number [28]. Seminal studies by Bauman and Kemper in the 1980s initially examined two cases and in the 1990s an additional seven autism brains that were embedded in celloidin and serially sectioned across a wide age range [2, 6, 29]. Qualitative observations reported that the most obvious PC decrease was in the posterolateral cerebellar cortex in the hemisphere with more normal observations in the medial cerebellar cortical regions such as the vermis. In 1998, Bailey and colleagues [5] examined six autism brains aged 4 to 27 years old compared to eight control cases aged 4 to 43 years and found that in 5 of 6 autism cases there were reduced numbers of PCs. In that study, a moderate decrease in PCs was seen in the hemisphere and vermis—it is noteworthy that 5 of 6 autism brains had a history of seizures. Four years later, another study examined the cerebellum in five autism and five control cases with a mean age of 25 years but counted Nissl sections from only 2 sections per brain and estimated that there was no statistical density difference between the two groups [30]. In two of the five cases, there was reported to be about a 50% smaller PC neuronal volume but overall the autism group was not significantly different from control. These authors reported more PC decreases in the seizure history case and patchy reductions in the nonseizure case. More recently, Whitney et al. [26] used stereological techniques to count the density of PCs in the posterolateral cerebellar hemisphere of six autism cases compared to five matched controls in young adults. In that study, three of the autism brains had a density of PCs in the normal range, two cases had mild/moderate PC decrease, and one had a severe PC decrease. The case with a severe PC decrease in the hemisphere did not have a PC decrease in the vermis, indicating a targeted pathology. That case also contained “empty baskets” which represents the persistence of inhibitory basket cells in the cerebellar molecular layer as evidenced with a silver stain whereas the other two cases with decreased PCs did not. This may give clues to the timing of the PC decrease in autism. Purkinje cells migrate from the ventricular zone in the roof of the 4th ventricle to reach their proper positions in the PC layer by about 30–32 weeks of gestation and subsequently elaborate their extensive planar dendritic tree in the molecular layer. There are little or no major disruptions in the layering of the cerebellar cortex, even in the hemispheres where the PC decrease appears to be the most impacted. It is therefore likely that PC migration occurred normally, thereby suggesting that the insult most likely occurred subsequent to 32 weeks prenatally, most likely persisting into early postnatal development. In the one case cited by Whitney et al. [26] with empty baskets, it is likely that the PC decrease occurred much later and may have been due to seizure or seizure medications but remains unclear.

Perhaps an unexpected finding came in 2004 when Vargas et al. [31] reported neuroglial activation and neuroinflammation in the cerebellum, anterior cingulate cortex, and the middle frontal gyrus in postmortem autism cases compared to age-matched controls. The neuroimmune responses were especially noteworthy in the cerebellum localized in the Purkinje and granule cell layers. Reactive astrogliosis was observed in both layers as well as microglia, macrophages, and specific types of cytokines such as MCP-1. Similar reactions were observed in the two cortical regions as well suggesting that neuroimmune reactions play a pathogenic role in ASD and may contribute to the diversity of autistic phenotypes [32]. 

Another interesting finding on cerebellar structure came in the early ‘90s by Courchesne and colleagues [33, 34] that reported, based on structural MRI findings, that there were two emerging subgroups of patients—one with a hypoplastic vermal lobules VI and VII and another subgroup with hyperplastic vermal lobules VI and VII. These authors also suggested that the onset of autism may be as early as the second trimester. It is left unclear, however, as a follow-up study by Schaefer et al. [35] determined that the finding of hypoplasia of vermal lobules VI and VII is a nonspecific finding that occurs in conditions without autism and may not be clinically useful as a predictive neuroanatomical marker for practitioners. Currently, there are studies using magnetic resonance spectroscopy that are being used to measure certain neurotransmitter levels and metabolites in specific brain areas. With the advent of high-resolution 3.0 Tesla machines, it is now possible to measure GABA, glutamate, glutamine, and others—manuscripts are beginning to emerge which will provide invaluable information that can be combined with postmortem findings to better interpret and localize abnormalities in these structures and possible relationships to core autism behavioral symptoms. 

### 2.2. Cholinergic Receptor Differences in the Cerebellum in ASD Patients

Elaine Perry and colleagues used cholinergic markers to determine whether the cerebellum and select cortical areas might contain abnormal levels of particular cholinergic receptor types [36, 37]. These authors reported a 40–50% reduction in nicotinic cholinergic receptor types *α*3, *α*4, and *β*2 measured by the high affinity agonist epibatidine in the granule cell, Purkinje cell, and molecular layers in cerebellum postmortem autism cases relative to matched controls. Perhaps a more striking result was a reported threefold increase in nicotinic cholinergic receptor type *α*7 in the granule cell layer measured by ligand binding using *α*-bungarotoxin. Nicotinic cholinergic receptor type *α*7 is localized to the surface of GABA inhibitory neurons and selective stimulation of *α*7 helps to trigger GABA release and thus play a role in restoring inhibitory tone. A subsequent study reported an increased *α*4mRNA expression in cerebellum but a reduction in subunit protein levels. It was suggested that there may be a relationship to reduced numbers of PCs in the autism cases but that remains unclear. The use of nicotinic cholinergic antagonists including some antidepressants has helped to ameliorate some autistic symptoms and these are being explored as novel therapeutic agents [38], whereas nicotinic agonists are utilized to enhance attentional processes, memory, and cognition and may also be useful in pharmacotherapies [39]. A summary of changes of additional neurotransmitter receptors and proteins in the cerebellum can be found in a recent review [40].

### 2.3. Inferior Olivary Complex Neuropathology in ASD

**Figure 1:**
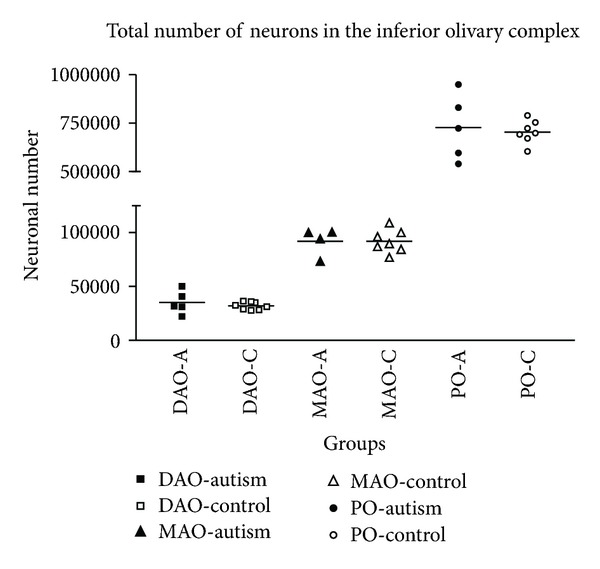
Scatter plot for neuronal number in the inferior olivary complex by group. No significant differences were found for the IOC (DAO: dorsal accessory olive; MAO: medial accessory olive; PO: principle olive). A: autism, C: control.

**Figure 2:**
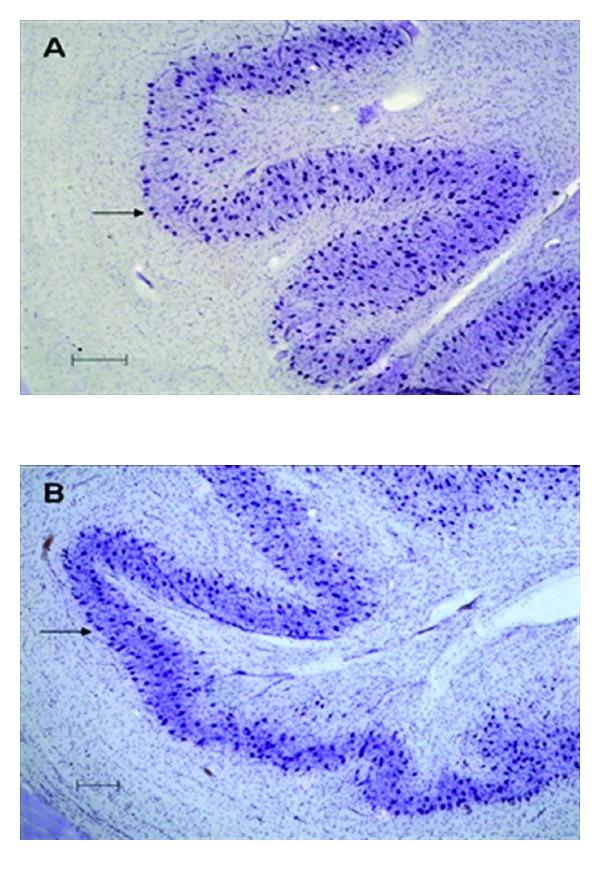
Photomicrographs of the inferior olive to show clustering at the edge of principle olive in an autistic brain. In A (autism) the clustering of neurons can be seen as a single row at the edge of the principle olive (arrow), whereas in B (control) these neurons are more scattered although some neurons do exist on the edge of the ribbon (arrow). Scale bars = 200 *μ*m.

**Figure 3:**
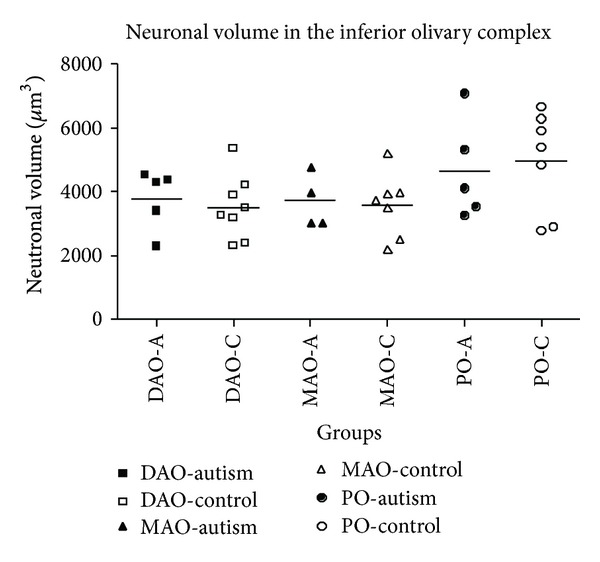
Scatter plot showing the perikaryal volume for the inferior olivary complex by group (DAO: dorsal accessory olive; MAO: medial accessory olive; PO: principle olive). Although there were no significant differences found, the PO neuronal volume was quite variable in both autistic and control brains. A: autism, C: control.

**Figure 4:**
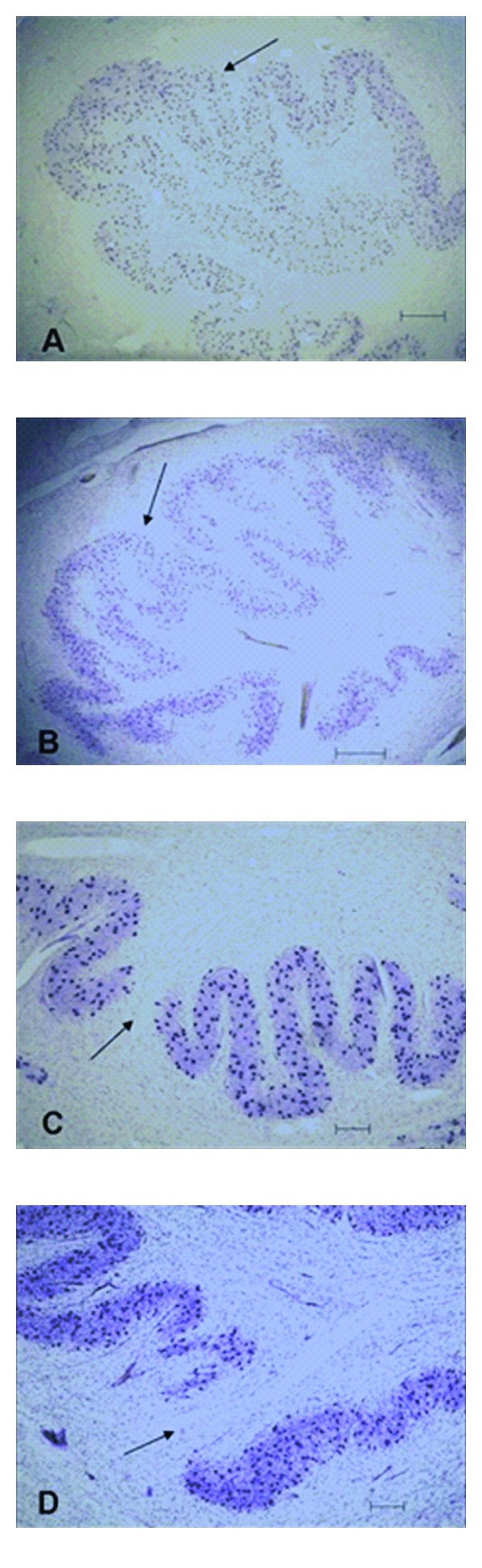
Photomicrographs showing similar altered formations of the principle olive in the autistic (A and C) and control brains (B and D). A and B show what appear to be reduplications (arrows) and C and D show examples of a gap in the ribbon (arrows) of the principle olive which may be caused by perforating vessel(s).

A brainstem structure that is intimately related to the cerebellum is the inferior olivary complex (IOC), which provides olivocerebellar climbing fibers directly to PC dendrites in the cerebellum [41–43]. Early qualitative studies in the postmortem autism brain reported that the part of the IOC (i.e., principal olive) related to the cerebellar hemisphere contained abnormally small, pale staining neurons without cell loss [2]. These authors also reported age-related differences with regard to olivary neuronal size and distribution within the nuclei such that in the adult (>22 years of age), the neurons are small in size, present in adequate numbers, and contained an abnormal peripheral distribution of neurons along the edge of the loop of the principal olive [3]. In contrast, neurons from an autistic child's principal olive were significantly larger than controls, but also present in adequate numbers and contained a similar abnormal distribution of neurons along the edge of the olivary ribbon [3]. Bailey et al. [5] examined 6 autism postmortem cases and reported a flattened medulla oblongata (contains the IOC and the pyramids) in one case and a large medulla with small pyramids in another case. These authors also reported “breaks” in the parts of the inferior olives in three cases, duplication of parts of the olivary ribbon and neuronal ectopia in four of the cases including neurons lateral to the olives in three cases and small groups of neurons in the inferior cerebellar peduncles in three cases [5]. These observations suggest that an additional insult likely occurs earlier than the PC decrease in a number of autism cases suggesting multiple gene effects on events that include neuronal migration and proper distribution of neurons. Bauman and Kemper [3] note that abnormal peripheral clusters of neurons in the IOC are also a pattern that has been reported in some syndromes of prenatal origin including mental retardation [44, 45]. Neuronal ectopia, similar to Bauman and Kemper's findings of abnormal distribution of IOC cells in the principal olive ribbon, was also found in additional postmortem cases from our laboratory as seen in Figure 1. In that same study, IOC cells were counted via stereological methods through the entire IOC in six autism cases and found that there were no significant differences in number in any of the three major nuclei (medial accessory olive, dorsal accessory olive, and principal olive) as seen in Figure 2, or in neuronal volume (Figure 3). This was a surprising finding because of the PC decreases that have been widely reported in the literature and the expectation would be retrograde IOC neuronal loss. It is likely, however, that sustaining collaterals to other PCs (up to 8–10 in the human) as well as to the deep cerebellar nuclei (DCN) might contribute to the survival of IOC neurons. Bauman and Kemper [3] hypothesized that the sustaining collaterals to the DCN represent the persistence of a primitive but adequately myelinated “fetal circuit” [46] to the DCN in adults originating at 28 weeks, a few weeks prior to their innervation to PCs. One feature of the IOC that turned out to be quite variable is the report of interruptions in the olivary ribbon. In studies from our laboratory, it was found that breaks and duplications in the structure of the ribbon can also be found in control cases (Figure 4) and may be influenced by perforating arteries and/or the three-dimensional structure folding over on itself due to the plane of section. Nevertheless, it appears to be more frequent in autism cases and may represent a discontinuity within particular olivary subnuclei.

### 2.4. Neuropathology of the Deep Cerebellar Nuclei in ASD

**Figure 5:**
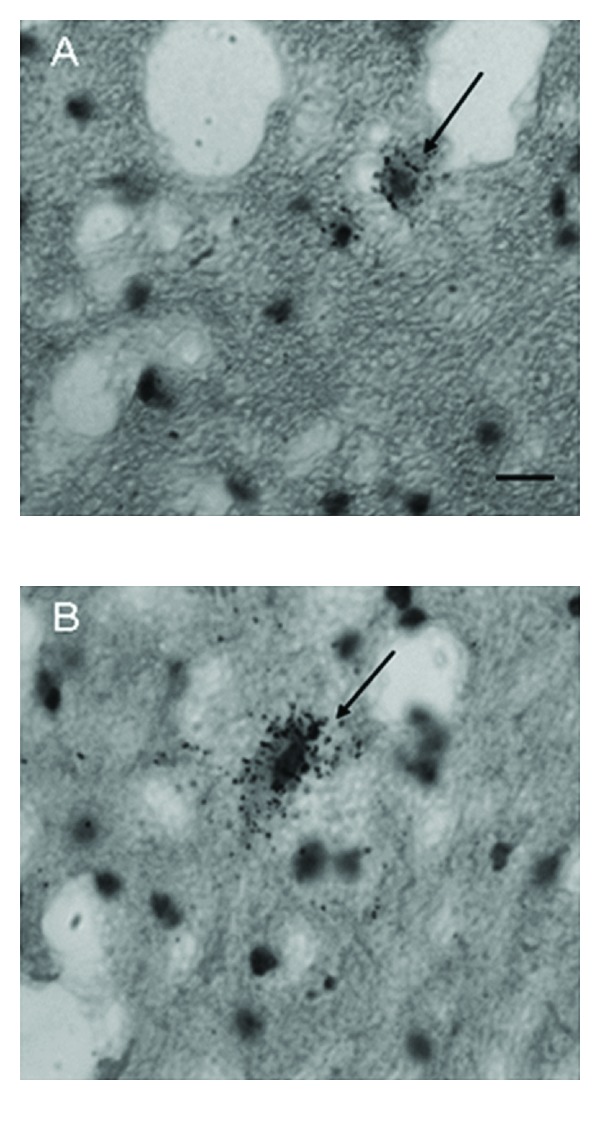
GAD65 mRNA-labeled neurons (arrows) in the dentate nuclei in a control case illustrating a small cell in A and a larger cell in B. Note the silver grain GAD65-mRNA-positive labeling throughout both cells. Scale bar in A = 20 *μ*m and refers to both A and B. Permissions granted from modified figure found in Yip et al., 2009 [47].

It is unknown to what degree the neurons of the DCN that are also impacted in autism as a quantitative study of the four nuclei (fastigial, globose, emboliform, and dentate) are lacking in the autism literature. However, in the celloidin fixed cases from Bauman and Kemper [2, 3, 6], a qualitative observation of a pallor of Nissl staining was observed in the granular layer of the hemisphere as well as the observed PC decrease and atrophy of the folia in the same region. The neurons of three of the DCN were small and pale and reduced in number (i.e., via qualitative observation) and the dentate nucleus was distorted in appearance, contained pale cells but were considered to be present in adequate numbers [3]. A more recent in situ hybridization study using a ^35^S probe directed toward glutamic acid decarboxylase type 65 (GAD 65) labeled two distinct neuronal populations within the dentate nucleus in adult autism and age-matched control cases [47]. The labeled GAD 65 was contained in two types of dentate neurons based on size—one population were about 10 um in diameter and the other about 20 um (Figure 5). The smaller population were assumed to be GABAergic interneurons that innervate other dentate neurons, and the larger population were likely those dentate neurons that project out of the cerebellum directly to the IOC as described previously in animal models [48]. It was this latter population of dentate neurons that contained significantly reduced GAD 65 indicating that the inhibitory control of the IOC may be impacted in autism. This is especially important due to the normally synchronous firing of populations of IOC neurons to PC targets [49]. With the reduction of PCs in autism coupled with abnormalities in the morphology of the DCN, ectopic IOC neurons within the principal olive ribbon, and presumably aberrant orientation of their dendrites, it increases the likelihood that there will be asynchronous activity of IOC neurons thus impacting the timing of PC firing and cerebellar output to higher structures [50, 51].

### 2.5. Brainstem Abnormalities in ASD

In a recent magnetic resonance imaging (MRI) study in 22 right-handed, non-mentally retarded boys with autism compared to 22 gender- and age-matched controls, a decrease in brainstem gray-matter volume was observed in the autism group with no significant differences observed in white-matter volume [52]. In that study, a significant relationship was observed between brainstem gray-matter volume and oral sensory sensitivity as measured by the Sensory Profile Questionnaire (SPQ). This study adds to the existing literature regarding abnormalities in the brainstem of autistic patients. Findings from postmortem IOC as well as imaging results need to be taken into perspective. About a decade ago, a published report by Rodier described major abnormalities in one female autistic patient including a missing “band” of tissue in the brainstem affecting a number of structures [53]. In that report, the author described the near absence of the facial nucleus, controlling muscles of facial expression, and the superior olive, a relay for auditory information, and postulated that since these two structures were from the same part of the embryo's neural tube, it may represent an early defect in autism, as early as 4 weeks prenatally. There were also differences in the rostrocaudal measurements between the trapezoid body (another auditory structure) and the lower brainstem structures including the hypoglossal nucleus and IOC. What has been found since that report has been more subtle, yet important findings of only specific brainstem structures as described above. In six brainstems obtained from The Autism Research Foundation (TARF) and The Autism Tissue Program (ATP), stereological analysis in our lab failed to demonstrate any significant differences in the locus coeruleus [54] or in the superior olive or facial nuclei [55]. The IOC was the only brainstem structure examined to demonstrate abnormalities in these cases yet clearly with multiple investigators' reports of IOC changes in the literature, there are forces at work in early prenatal development. Genetic findings affecting this brain region in autism include the Homeobox A1 (HOXA1) gene which has been proposed as a candidate gene for ASD for over a decade, as it regulates embryological patterning of hind-brain structures implicated in autism neurobiology [56]. The authors suggest that common genetic variation within this putative ASD risk gene has the capacity to modify the development of cerebellar systems and thus may in part play a role in brainstem abnormalities observed in ASD. 

## 3. Limbic System Abnormalities in ASD

### 3.1. Hippocampus

 As early as 1985, abnormalities were noted in the hippocampus in postmortem patients with autism [2]. In two brains that were serially sectioned, the Nissl stained hippocampus qualitatively demonstrated increased cell packing density (increased numbers of neurons per unit volume) throughout the CA and subicular subfields. The increased cell packing density in the hilar CA4 subfield gave the structure a more flattened appearance when compared to controls. In a later report when more cases were included in the studies, Bauman and Kemper [3] also reported reduced pyramidal cell size in the hippocampus in autism cases. A Golgi analysis of the CA1 and CA4 regions revealed a decreased complexity and extent of dendritic arbors [57]. Stunting of the dendritic arbors as well as reduced secondary and tertiary branching in these CA neurons were demonstrated. In a follow-up study, Lawrence et al. [58] investigated whether there might be alterations in the density of hippocampal GABAergic neurons in postmortem autism cases. These investigators used stereological techniques and immunocytochemical methods to stain three types of calcium binding proteins to label subpopulations of GABAergic interneurons in the hippocampus in five autistic cases and five age-, gender, and postmortem interval-matched controls. Quantitative results indicated a selective reduction in the density of calbindin-immunoreactive neurons in the dentate gyrus (including hilar CA4), an increased density of calretinin-immunoreactive neurons in the CA1 region, and an increased density of parvalbumin-immunoreactive neurons in areas CA1 and CA3 in the autism cases compared to controls. Together, the neuropathological and immunocytochemical studies demonstrate a selective vulnerability of populations in hippocampal pyramidal and interneurons thus suggesting that this key limbic structure, that plays an important role in learning and memory, especially in declarative memory [59], including the ability to retain and recall episodic memories, is impacted in autism. 

### 3.2. Entorhinal Cortex

The formation of new declarative memories relies on both the hippocampus and parahippocampus [60] and it is interesting the anterior most part of the parahippocampal gyrus, the entorhinal cortex (Brodmann area 28), also has demonstrated abnormalities in autism. In postmortem cases, it was found that a relatively clear zone deep to the superficial layer, the lamina dissecans, persists in the adult autism cases, whereas in control cases, it typically disappears during childhood [2].

### 3.3. Amygdala

Pathology in the amygdala was also reported in the early studies by Bauman and Kemper. Similar to the hippocampus, it was observed that there was an increased packing density in select amygdalar subregions including the central, medial, and cortical nuclei by about 30–35% with reduced cell size also reported in these regions [2]. The basolateral complex was noted to have only minor changes. Follow-up studies were conducted by Schumann and Amaral [61] who first used stereology and the fractionator method in ten human postmortem cases through the entire amygdala and all of its nuclear regions to establish baseline counts upon which to subsequently compare cases with neurological disorders such as autism. Then in the first quantitative stereological study of the autistic brain, neurons in several amygdala subdivisions of nine autism male brains were counted and measured and compared to the counts from ten age-matched male control brains [62]. In that study, there was no difference in the overall volume of the amygdala or in individual subdivisions nor were there any changes in cell size. The authors did report that there were significantly fewer neurons in the autistic amygdala overall and in its lateral nucleus. These results when combined with structural MRI studies suggest that the autistic amygdala appears to undergo an abnormal growth pattern of postnatal development that includes early enlargement and ultimately a reduced number of neurons [63, 64]. Interestingly, children with autism aged 7.5–12.5 years of age had larger right and left amygdalar volumes when compared to typically developing control children but there were no differences in amygdala volume between the adolescent groups (12.75–18.5 years of age). Since the amygdala in typically developing children increases substantially in volume from 7.5 to 18.5 years of age, the authors concluded that the amygdala in children with autism is initially larger, but does not undergo the age-related increase observed in typically developing children [64].

### 3.4. Anterior Cingulate Cortex (ACC; Brodmann Area 24)

The anterior cingulate cortex (ACC; Brodmann area 24 or BA 24) participates in a variety of functions including executive, evaluative, and cognitive functions and emotion [65–67]. Information processing of high-order sensory and multimodal information occurs via its connections with the prefrontal cortex and parietal cortex and with the motor systems, including the frontal eye fields [68–72]. The ACC participates in early learning and problem solving [68], as well as error detection, emotional response to pain, anticipation of tasks, motivation, monitoring of social interaction, and modulation of social-emotional responses [73–76]. It has also been postulated that the ACC plays a role in theory-of-mind due to circuitry linking the ACC with the adjacent frontal cortex and temporoparietal junction [77]. This circuit may also be involved in joint attention that is deficient in many individuals with autism [78, 79]. Thus, disruptions of this region, at any level, cellular or chemical, could lead to social, communication, and other behavioral deficits. 

**Figure 6:**
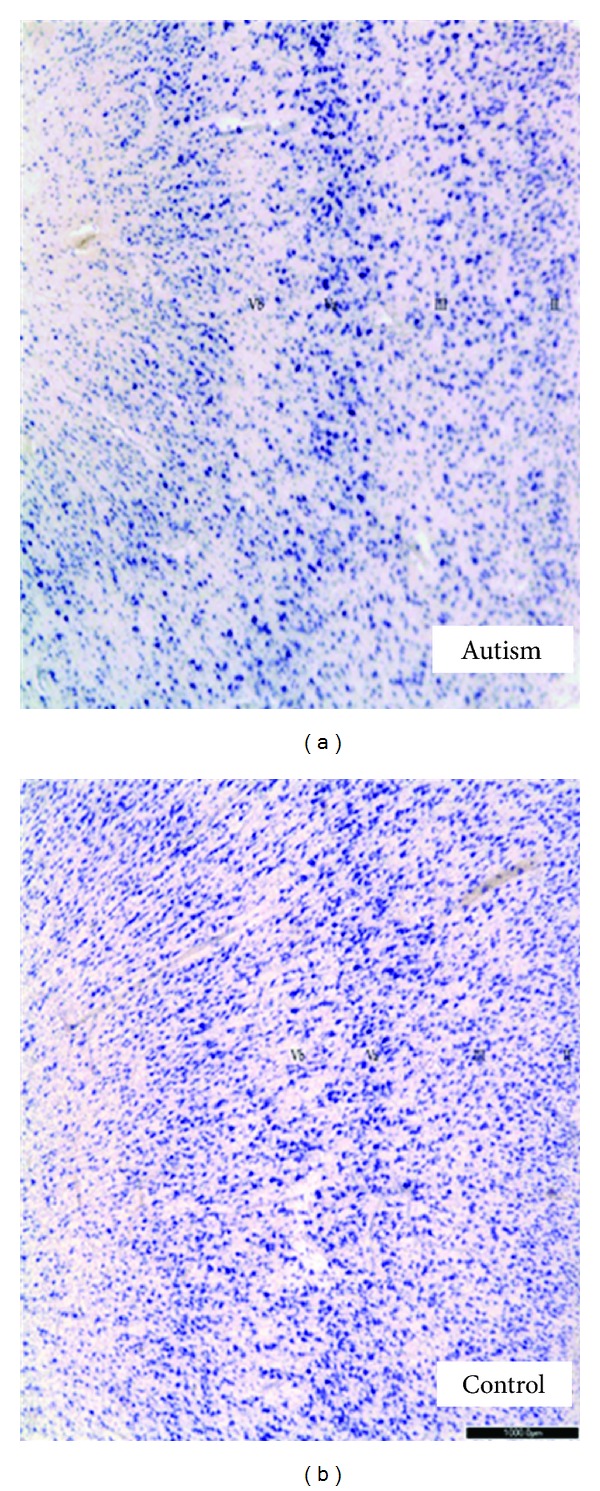
Lamination of ACC in control and autism brains (40x). Note irregular lamination obvious in layers III-V in an autism case (left figure) compared to control at right. In each figure, layer II is on right side, layers V-Vi at left. Also note, the ACC lacks a layer IV. Permissions granted for modified figure from Simms et al., 2009 [81]. Scale bar = 1 mm.

Bauman and Kemper first observed qualitatively cytoarchitectonic differences in the anterior cingulate cortex in their early autism cases despite much of the cerebral cortex being observed as relatively unremarkable [2]. These authors also reported that the ACC in autism cases was poorly laminated with small cells and altered packing density [6, 29, 80]. In a subsequent study, a significant decrease in neuronal area was observed in the supragranular and infragranular layers of area 24b (a subregion of the ACC) as well as a significant decrease in cell volume in the supragranular and infragranular layers of the same region [81]. Specifically, there was a significant decrease in neuronal size in layers I–III and layers V-VI in area 24b and in cell packing density in layers V-VI of area 24c. Cytoarchitectonic abnormalities were seen in 3 of 9 autism cases; an example is found in Figure 6. 

Increased density of neurons in the white matter adjacent to layer VI was present in the remaining cases suggesting developmental anomalies [81]. The ectopic distribution of neurons within specific lamina of the ACC represents abnormal distribution within the target lamina due to a failure of proper migration to the cortical plate [82]. The increased neurons in the white matter in autism cases may be due to a persistence of a normally transient fetal/neonatal primordial plexiform layer, a recipient region for neuronal migration that separates it into layer I, and a transient subplate zone deep to the cerebral cortex [83, 84]. The excess neurons in the white matter in autism cases in the region of the ACC may represent a lack of completed migration from the subplate into the primordial plexiform layer thus affecting subcortical and cortical circuitry [85–88]. Collectively, these abnormalities in neuronal migration represent early developmental defects in the autism brain. Interestingly, similar cytoarchitectonic abnormalities as well as white matter neuron increases were also noted in the posterior cingulate cortex by Oblak et al. [89] discussed in the next section. It is also noteworthy that Bailey et al. [5] also reported atypical laminar patterns in the frontal cortex in postmortem autism cases.

Neurochemical differences in the ACC have been reported via imaging studies as well as from analyses from postmortem tissue samples from autistic patients. An imaging study by Murphy et al. [90] in eight adult subjects with Asperger's syndrome, using single-photon-emission computed tomography (SPECT) imaging, demonstrated a significant reduction in 5-HT2A receptor binding in select cortical areas including the anterior and posterior cingulate cortices and parts of the parietal and temporal lobes compared to ten matched controls. Interestingly, the decreased receptor binding was significantly related to abnormal social communication scores. It is unclear whether the 5-HT2A receptor changes might be related to the neuropathology within the cortical lamina or whether it is a more global effect since it was demonstrated in a number of brain regions. A recent study by Azmitia et al. [91] found increased 5-HT axons in the cortex of individuals with autism, suggesting that increased release of serotonin from these terminals may in part account for the reduced 5-HT2A receptors. In some cases, these axons were of a dysmorphic nature. Nakamura et al. [92] used PET imaging and found reduced 5-HT transporter (5-HTT) binding in the anterior and posterior cingulate cortices associated with impairment in social cognition in individuals with high functioning autism and reduction of 5-HTT binding in the thalamus correlated to obsessive behaviors and interests. Goldberg et al. [93] published findings from a PET imaging study on the parents of children with Autism Spectrum Disorders (ASD) having significantly reduced 5-HT2 binding and found that their platelet 5-HT levels were inversely correlated to the 5-HT2 binding potential. 

In addition to changes in serotonin receptors, alterations in GABA receptors have also been reported in the ACC in adult autism patients from postmortem tissue analyses. Oblak et al. [94] studied the benzodiazepine binding site on the GABA_A_ receptor complex due to its importance as a target for pharmacotherapy and its clinical implications. The multiple-concentration ligand-binding study utilized ^3^H-flunitrazepam to determine the number (*B*_max⁡_), binding affinity (*K*_*d*_), and distribution of benzodiazepine binding sites and used ^3^H-muscimol to label GABA_A_ receptors in the ACC in adult autistic and control cases. The autistic group had significant decreases in the mean number of benzodiazepine binding sites in the supragranular (28.9%) and infragranular (16.4%) lamina and in the number of GABA_A_ receptors in the supragranular (46.8%) and infragranular (20.2%) layers of the ACC. In addition, a trend for a decrease in for the number of benzodiazepine sites was found in the infragranular layers (17.1%) in the autism group suggesting that this downregulation of both benzodiazepine sites and GABA_A_ receptors in the ACC may be the result of increased GABA innervation and/or release disturbing the delicate excitation/inhibition balance of principal neurons as well as their output to key limbic cortical targets. It is unknown whether the excitatory components, that is, glutamate and glutamate receptor subtypes, are also changed in the autism group in the ACC but experiments are currently underway aimed to address this issue. 

In another study, Oblak et al. [95] also looked at GABA_B_ receptors in the ACC in adult autism cases relative to matched controls. GABA_B_ receptors play an important role in modulating synapses and maintaining the balance of excitation/inhibition in the brain. The density of GABA_B_ receptors in subjects with autism and matched controls was quantified in the anterior cingulate cortex and significant reductions were demonstrated. The authors postulate that alterations in this key inhibitory receptor subtype may in part contribute to the functional deficits in individuals with autism. Interestingly, the presence of seizure in a subset of autism cases did not have a significant effect on the density of GABA_B_ receptors in the ACC. Oblak et al. [95] found similar results in two other cortical regions, the posterior cingulate cortex (PCC) and the fusiform gyrus (FG) discussed below.

### 3.5. Posterior Cingulate Cortex (PCC; BA 23)

The PCC in postmortem autism cases also has laminar abnormalities that are relatively similar to the ACC but had more extensive findings. Oblak et al. [96] examined 8 autism cases via Nissl stained sections from tissue blocks obtained from brain banks. In that study, one case had increased neurons in layer I, and increased cell packing density in layer III. Another case had large neurons with irregular distribution in layer II but smaller neurons distributed in layer III and irregular distribution of neurons in layer V. Four other cases had layer V abnormalities and three cases had increased density of white matter neurons similar to findings in the ACC. These cortical abnormalities represent a failure of normal cortical development with respect to the migration of neurons from the ventricular germinal zone to the cortical plate which occurs between 8 and 22 weeks of gestation [82, 88]. Interestingly, in the same 8 cases, GABAergic interneuron subpopulations were counted via immunocytochemical techniques. Both parvalbumin-immunoreactive neuronal density and calbindin-immunoreactive neuronal density were preserved in the PCC in the autism cases relative to matched controls. This finding is surprising because as previously noted, Lawrence et al. [58] did find subfield selective significant changes in interneuron density in the hippocampus in autism cases. It is also a key finding because in two other studies, Oblak et al. [94, 95] using ligand binding techniques noted a quantitative reduction in both benzodiazepine binding sites and GABA_A_ receptors in the PCC in adult autism cases as well as a reduction in the relative density of GABA_B_ receptors in the same region. Taken together, it is likely that the density of GABAergic innervation to pyramidal cells in the PCC is altered due to either altered GABA release or due to changes in the recipient dendritic arbors. This is quite interesting in light of the fact that Oblak et al. [96] used stereological techniques to quantify GABAergic interneurons in the PCC from calbindin and parvalbumin immunolabeled subpopulations (two of the most common types) and found that there were no differences in relative density in the supra- or infragranular lamina in the young adult ASD group (*n* = 7-8) compared to matched controls (*n* = 7). There was a statistical trend for reduced calbindin-immunoreactive neurons in both the superficial and deep layers of the PCC and this will need to be followed up by additional studies with increased numbers of ASD cases. 

### 3.6. Defects in the Default Network in ASD

The functional significance of defects in the cingulate cortices in autism is that they play a critical role in developmental processes, formation of circuits, and presumably in the disruption of the excitatory/inhibitory balance within the gyrus. It is likely that such disturbances, with a prenatal origin, will affect behavioral domains in autistic patients in a variety of ways. Certainly social-emotional and social-communicative behaviors come into play and it has already been shown that differences in neurochemical density and distribution can be related to such behaviors. But often overlooked is another key aspect of the both cingulate areas: their critical roles in the default mode network (DMN), a network that is active during passive resting states and cognitive processes and has been shown to be defective in autism [97–100]. Of interest in autism is how such alterations in DMN might relate to social deficits in ASD such as Theory of Mind. Assaf et al. [99] used data-driven analyses of DMN subnetworks they term DM-SNs, in sixteen high functioning ASD patients compared to sixteen typically developing controls. The authors concluded that their analyses supported the hypothesis that DM-SNs underconnectivity of the precuneus with the frontal and anterior cingulate cortices contributed to the core deficits in ASD based on the Social Responsiveness Scale and Autism Diagnostic Observational Schedule (ADOS). Monk et al. [98] describe the default network to include the posterior cingulate cortex, retrosplenial cortex, lateral parietal cortex/angular gyrus, medial prefrontal cortex, superior frontal gyrus, temporal lobe, and parahippocampal gyrus, all strongly active when there is no task being performed. Using fMRI, these authors found that poorer social functioning in the ASD group was correlated with weaker connectivity between the frontal cortex and PCC. In contrast, more severe repetitive behaviors and restricted interests in the ASD patients were correlated with stronger connectivity between the right parahippocampal gyrus and the PCC. Therefore, within the default network, there is a disturbance in the intrinsic connectivity and between DMN structures that participate in key ASD behaviors, including both the ACC and PCC. 

## 4. Neocortical Pathology in ASD

### 4.1. Cortical Dendritic Abnormalities in ASD

Hutsler and Zhang [101] used the Golgi method and recently found alterations in dendritic spine densities on cortical projection neurons in ASD. These authors examined portions of the temporal, parietal, and frontal lobes and found increased dendritic spine densities in layer II in all three cortical regions and in layer V of temporal lobe cortex. Higher spine densities were also correlated with decreased brain weights and commonly found in cognitively impaired ASD subjects. These authors discuss the possibility that the increased spine densities may be the result of deficient culling of connections during the postnatal period and may represent an alteration of the density of excitatory synapses to these cortical neurons. These results make an important contribution to the literature and further expand on cortical changes in ASD that have been reported over the last decade.

### 4.2. Aberrant Organization of Cortical Minicolumns in ASD

**Figure 7:**
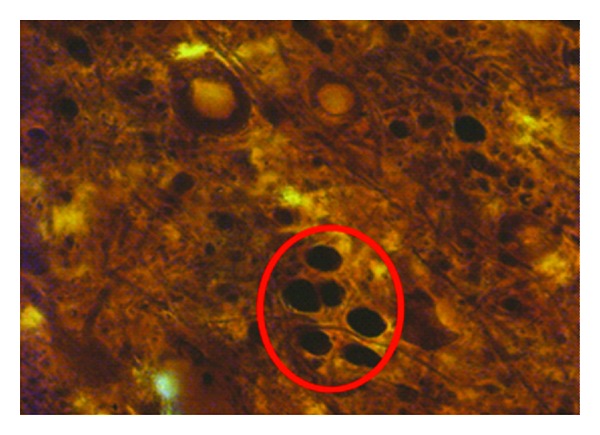
A 50 *μ*m vibratome cut section demonstrates vertical minicolumnar organization in the human inferior frontal cortex via immunohistochemistry section incubated with primary antibodies to microtubule-associated protein type 2 (MAP2) that labels apical dendrites (Figure 1; 1000x; red circle). Note the radial organization of columnar components in this tangential section through layer IV. This represents the center of a cortical minicolumn.

Perhaps one of the most striking findings over that time period has been the series of articles by Casanova and colleagues initially reporting that in layer III of prefrontal and temporal cortex there is an alteration in cell columns in ASD [102, 103]. These investigators reported that there was an increased number of cortical “minicolumns,” combined with fewer neurons per column and less neuropil space in their periphery ([103] summarized in [104]). Minicolumns are functional modular arrangements of neurons that span most or all neocortical layers of the brain and serve to organize neurons in a defined space and have similar response properties [105, 106]. They are part of a larger macrocolumnar system which is thought to be important in generalization whereas smaller minicolumns may facilitate discrimination [103, 107]. The smaller peripheral neuropil space of minicolumns in ASD has hypothesized impact GABAergic innervation to the minicolumnar neurons and thus may interfere with signal processing and differentiation [103], but further studies are needed. In this light, DeFelipe et al. [108] and subsequently Peters and Sethares [109] had previously described the presence and role of immunocytochemically stained double bouquet cells and how they relate to pyramidal cell modules in the monkey striate cortex. These GABAergic interneurons have vertically oriented “horse-tail” axons arranged in a regular array in layers II/III such that they run alongside pyramidal cell dendritic clusters, whereas in layer IV_C_ they are closely associated with myelinated axon bundles [109]. It would be quite interesting to determine whether there are alterations in the density or distribution of double-bouquet cells in autism and how they might relate to increased number and narrower minicolumns. Internal components of minicolumns can also be observed by using antibodies to microtubule-associated protein type 2 (MAP2) that labels apical dendrites. An example of a tangential section cut across layer IV in human frontal cortex is seen in Figure 7. It represents an alternate method to view and quantify minicolumnar organization but is a very difficult and time-consuming approach. 

The actual technique of quantitative analysis of minicolumns was described by Schlaug et al. [110] in the Zilles lab in Dusseldorf. The index of verticality describes the deviation of a distinct brain area and layer(s) from the mean degree of vertical organization (based on Nissl-stained material) and all layers examined. In short, different degrees of columnar organization can be quantitatively described by the verticality index and used as criteria to describe and characterize different cerebral cortical areas [110]. These methods were modified and updated by Buxhoeveden et al. [106, 111] and utilized in their studies on ASD Nissl stained material. Of interest are Gustafsson's comments that narrower minicolumnar organization in autism may, alternatively, be due to an early low capacity for producing serotonin (5-HT) as described in autism [112, 113] as such alterations in minicolumn structure have been seen in lab animals with lower 5-HT levels. Another alternative hypothesis is due to insufficient nitric oxide, which has been shown from neural network analysis to cause narrower neural columns [107]. More recently, Casanova and colleagues conducted a comprehensive analysis of Nissl-based photo mosaics from a number of cerebral cortical areas in autism [114]. The greatest difference between autism (*n* = 7; aged 4–67 years) and control (*n* = 7; aged 4–65 years) groups in the study was observed in Broca's area (Brodmann area 44) with narrow minicolumnar organization similar to that found in other select cortical regions in ASD. 

### 4.3. Abnormal Frontal Lobe Growth in ASD

Behavioral symptoms in ASD first appear with subtle abnormalities in motor, sensory, attention, and social behavioral and appear as early as the first or second year of life [115–119]. By the time an affected individual is about 2 to 3 years of age, there are failures to achieve normal language and social developmental milestones leading to proper diagnosis of ASD [119]. It has been known for a decade that abnormalities in brain growth, including enlargements of cerebral, cerebellar, and limbic structures, occur in subjects with ASD at about this age range [63, 120–122]. This corresponds to the observable symptomatology of ASD and subsequent observations of reversed asymmetry in frontal language cortex in boys with ASD [123–125]. Interestingly, the period of brain overgrowth is followed by an abnormally reduced rate of brain growth [119]. The abnormal cortical overgrowth is especially pronounced in the frontal lobes, particularly medial and dorsolateral regions, and has been hypothesized to affect the developmental timetable for synaptogenesis, dendritic growth, and circuit formation with affects in higher-order social communication, emotional processing, language, and cognition [119]. 

### 4.4. Fusiform Gyrus (BA 37) Neuropathology in ASD

**Figure 8:**
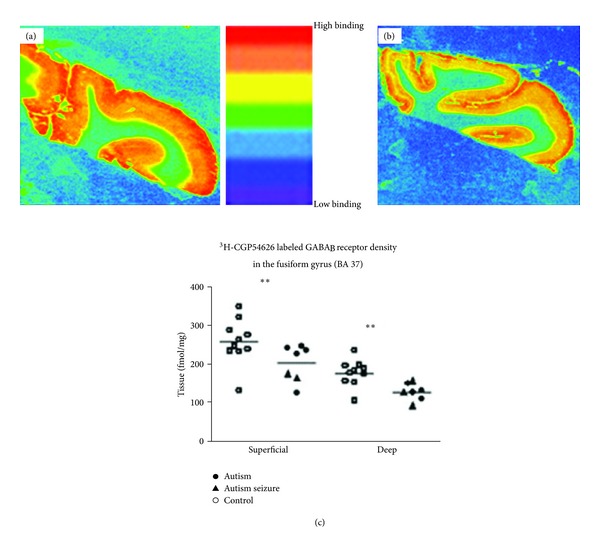
Graph demonstrating [^3^H]-CGP54626 labeled GABA_B_ receptor binding density in the fusiform gyrus (FG) from a control (a) and an autistic case (b). (c) is a scatter plot of all cases included in the study. Significant reductions (∗∗) in the superficial (*P* = 0.019) and deep (*P* = 0.00095) layers were found in the autism cases compared to age- and postmortem interval-matched controls. Permissions granted from Oblak et al., 2010 [95].

The FG, also called the occipitotemporal gyrus, extends the length of the inferior occipitotemporal region between the collateral sulcus and parahippocampal gyrus (medially) and the occipitotemporal sulcus (laterally) [126] and is differentiated from these structures based on differences in cytoarchitecture [127]. There are two published reports that quantify neurons in the fusiform gyrus (FG) in the temporal lobe, a region that contains the fusiform face area (FFA) and is important for identifying faces, perception of facial expressions, and emotional responses related to facial features and has shown to be altered (i.e., mainly hypoactive in fMRI studies) in ASD. The seminal study of the neuropathology of the FG was conducted by van Kooten et al. [128]. Using stereological techniques, these investigators found in seven postmortem brains from patients with autism compared to ten matched controls that there was a significant reduction in neuron densities in layer III in the FG, reduction in total neuron numbers in layers III, V and VI, and in the mean perikaryal volumes of FG neurons in layers V, and VI. These findings demonstrate that there are developmental abnormalities in the FG with regard to the density, number, and distribution of neurons in particular supra- and infragranular lamina and potentially could disturb information processing related to the ability to properly perceive facial expressions [128]. The second report used blocks of tissue from the FG from nine autistic patients aged 14–37 years compared to matched controls aged 16–36 years [89]. In marked contrast to the van Kooten study, no significant differences were noted in the densities of thionin-stained neurons, or in parvalbumin- or calbindin-immunoreactive interneurons in the FG. In addition, unlike the ACC and PCC, no cytoarchitectonic abnormalities in gray or white matter were observed. However, these same authors did find significant reductions in superficial (I–IV) and deep (V-VI) in ^3^H-flunitrazepam labeled benzodiazepine binding sites in the FG and significantly reduced ^3^H-muscimol labeled GABA_A_ receptor number in superficial layers in a multiple concentration ligand binding study [95]. In a previous study, Oblak et al. [95] also demonstrated significantly decreased ^3^H-CGP54626 labeled GABA_B_ receptor density in both superficial and deep layers in a single-concentration receptor binding investigation (Figure 8). It is not known whether these differences might be due to the changes in neuron number and/or cytoarchitecture such as reported by van Kooten et al. [128] but does infer that there are again differences in GABAergic innervation to pyramidal neurons in the FG despite relatively normal GABAergic interneuron densities throughout the FG lamina. Experiments are currently underway in our laboratory investigating the glutamatergic system in the FG, ACC, PCC, cerebellum, and in selective speech and language areas to gain a better understanding of how the excitatory/inhibitory balance might be disturbed in cerebellar, limbic, and/or neocortical regions in ASD.

## 5. GABAergic Pharmacotherapy in Autism: Targeting Binding Sites

Oswald and Sonenklar [129] reported that in 2002, 4.2% of autistic patients received at least one prescription for a benzodiazepine; there have been relatively few clinical studies of the efficacy of this class of agents in autism populations. This is quite interesting as evidence suggests that the density of benzodiazepine sensitive GABA_A_ receptors is reduced in autism [94, 130, 131]. One early case report describes paradoxical angiogenic and aggressive responses to diazepam in seven children with autism [132]; however, a later report describes the routine use of midazolam for preanesthetic sedation of autistic patients [133]. It is noteworthy that clinical trials are now addressing the likely excitation : inhibition imbalance in autism using metabotropic glutamate (mGluR) antagonists as well as GABAergic agonists for the treatment of ASD [134]. Both have promising preliminary data from animal studies and small scale Phase II human trials with favorable side effects and are awaiting larger scale double-blind placebo-controlled clinical studies. Recent findings from our laboratory have found increased mGluR1 mRNA in the cerebellum in adult individuals with autism in a postmortem study adding further evidence to the imbalance [135]. This is in contrast to recent findings regarding Selective Serotonin Reuptake Inhibitors (SSRIs) that have recently been shown to be largely ineffective in many individuals with autism with largely unfavorable and even harmful side effects [136]. 

## 6. Conclusions

Autism is a complex neurological disorder that has distinct neuropathological brain areas that are associated with core behavioral features due to their known functional roles. There is wide diversity in features among cases but there are also consistent findings that will undoubtedly contribute to the establishment of autism subgroups based on their structural, neurochemical, and genetic features. It remains to be determined whether blueprints will emerge that defines specific subgroups with predictive value for interventions and improved treatments. But strides are being made in a variety of disciplines aimed to distinguish such groups. Postmortem analyses have been largely based on a relatively small number of cases, and many with seizures in their clinical histories, as Amaral et al. [17] point out in their review. But despite this, consistent reliable findings have emerged with significant results, especially with regard to GABAergic receptor subtypes which take a major hit in the disorder. There are also many clues as to the timing of origin of ASD. Changes in the cytoarchitecture of limbic and neocortical areas and the presence of excess cells in the white matter adjacent to layer VI in the cingulate cortices give clues toward the late prenatal/early postnatal periods. Hindbrain changes such as a misalignment of inferior olivary neurons along the edge of the principal olivary ribbon of neurons suggest a prenatal insult, perhaps even in the 1st trimester in a subset of cases, and that is the key word, “subsets,” because it is very clear that values within the autism group of cases separate into those values that overlap with normal results and those that are found either up- or down-regulated. As the brain banks improve their collection of medical histories open to investigators as well as genetic background, additional patterns will emerge. Improved technologies in imaging are also leading the way toward determining not only structural and functional differences among brain structures, but also neurochemical profiles for both children and adults with the disorder. Certainly the genetic, epigenetic, and environmental influences will also lead the way toward a better understanding of why gene(s) are dysregulated in the autism brain and where they are expressed, and how this may correlate with imaging and neuropathological findings including neuroimmune changes. So we have come a long way but there is much more to explore and meanwhile, the incidence keeps rising. With the advent of DSM-5, the direction may change somewhat as different types of autism will be grouped as one, ASD, with what appears to be more stringent criteria. This may prove difficult to those that conduct postmortem research so the greater information that is gathered and made available to investigators, coupled with more cases donated, will propel the field toward a better mechanistic understanding of the disorder.
